# Insights on Platelet-Derived Growth Factor Receptor α-Positive Interstitial Cells in the Male Reproductive Tract

**DOI:** 10.3390/ijms25074128

**Published:** 2024-04-08

**Authors:** Tasuku Hiroshige, Kei-Ichiro Uemura, Kei-Ichiro Nakamura, Tsukasa Igawa

**Affiliations:** 1 Department of Urology, Kurume University School of Medicine, Kurume 830-0011, Japan; 2 Cognitive and Molecular Research Institute of Brain Diseases, Kurume University School of Medicine, Kurume 830-0011, Japan

**Keywords:** PDGFRα, interstitial cells, three-dimensional analysis, FIB/SEM, male reproductive tracts

## Abstract

Male infertility is a significant factor in approximately half of all infertility cases and is marked by a decreased sperm count and motility. A decreased sperm count is caused by not only a decreased production of sperm but also decreased numbers successfully passing through the male reproductive tract. Smooth muscle movement may play an important role in sperm transport in the male reproductive tract; thus, understanding the mechanism of this movement is necessary to elucidate the cause of sperm transport disorder. Recent studies have highlighted the presence of platelet-derived growth factor receptor α (PDGFRα)-positive interstitial cells (PICs) in various smooth muscle organs. Although research is ongoing, PICs in the male reproductive tract may be involved in the regulation of smooth muscle movement, as they are in other smooth muscle organs. This review summarizes the findings to date on PICs in male reproductive organs. Further exploration of the structural, functional, and molecular characteristics of PICs could provide valuable insights into the pathogenesis of male infertility and potentially lead to new therapeutic approaches.

## 1. Introduction

The issue of infertility in the developed world has reached a level where it is now considered a social concern. Male infertility constitutes a significant portion, with 20–30% of all infertility cases attributed to male factors [1]. These factors encompass hormonal disorders, physical ailments, lifestyle considerations, psychological issues, sexual problems, chromosomal abnormalities, and single-gene defects [1,2,3]. The primary causes of male infertility are a decreased sperm count and motility. A decline in sperm count may indicate not only reduced sperm production but also impaired transport through the male reproductive tract. Currently, there are no clinical or experimental measurements of sperm transport failure, and only limited objective evidence regarding the mechanisms of sperm transport has been documented. Consequently, many patients with unexplained low sperm count (oligospermia) may experience some degree of transport failure rather than spermatogenesis.

Sperm transport entails various factors, including sperm motility, smooth muscle contractions of the male reproductive tract, and seminal fluid dynamics [4,5]. Spermatozoa within the testes exhibit minimal motility upon ejaculation into the head of the epididymis but acquire motility during transit to the tail of the epididymis [6,7,8], a phenomenon termed sperm maturation. While mammalian sperm become motile post-ejaculation into the female genital tract, those attaining motility after traversing the epididymis remain inhibited within the male reproductive tract. Consequently, inactive sperm necessitate transportation from the seminiferous tubules to the rete testis via the efferent duct and epididymis to the vas deferens.

The mechanisms governing sperm transport in male reproductive organs have been the subject of various theories, predominantly implicating smooth muscle movement [9,10,11]. Understanding smooth muscle activity in the male genitalia is crucial for elucidating sperm transport mechanisms, potentially paving the way for male infertility treatment or the development of male contraceptives.

Smooth muscles within the male reproductive tract contract rhythmically to propel sperm along the tract. Their intrinsic activity ensures sperm movement even without neural input, underscoring the redundancy and resilience of the system [12]. While the autonomic nervous system does not directly control rhythmic contractions for sperm propulsion, it does modulate and coordinate these contractions alongside other critical functions throughout the sexual response [13]. Traditionally, discussions on smooth muscle movement have centered on the nerve–smooth muscle relationship. However, in smooth muscle organs such as the gastrointestinal tract and bladder, interstitial cells may interact with nerves to regulate smooth muscle contraction, with implications for various diseases [14,15,16,17,18]. This review focuses on the interplay between stromal cells and smooth muscle movement in the male reproductive tract, exploring the potential of interstitial cell research in this realm.

## 2. Interstitial Cells Involved in Smooth Muscle Movement

Functional studies of interstitial cells in the male reproductive organs often prioritize Sertoli and Leydig cells, which are associated with spermatogenesis. However, few studies have elucidated the functional relationship between smooth muscle movement and interstitial cells in the male reproductive tract [19].

Interstitial cells of Cajal (ICCs) and platelet-derived growth factor receptor α (PDGFRα)-positive interstitial cells (PICs) are two types of interstitial cells involved in smooth muscle movement, particularly in the gastrointestinal tract and bladder [20,21]. ICCs are renowned for their role in generating pacemaker activity and neurotransmission in the gut, while PICs have been implicated in bladder function and neuromodulation [18,22,23]. Both cell types are electrically coupled to smooth muscle cells (SMCs), forming a functional syncytium that regulates smooth muscle excitability [24].

ICCs, discovered by the Spanish neuroanatomist Santiago Ramón y Cajal, are present around the intermuscular plexus and in the circular muscle layer of the gastrointestinal tract [25]. They have been identified in various parts of the gastrointestinal tract, including the esophagus, stomach, cecum, and small intestine [26,27]. ICCs are thought to act as pacemakers in intestinal peristalsis. ICCs act as electrical pacemakers and generate waves known as slow waves, exhibiting periodic potential fluctuations [28,29]. These slow waves determine the rhythm of spontaneous contractions in the digestive tract. Slow waves originate from ICCs and propagate through gap junctions to surrounding ICCs and smooth muscle cells [30,31]. These slow waves cause an increase in membrane potential, leading to the influx of calcium ions and subsequent muscle cell contraction [32]. The propagation of slow waves depends on IP3 receptors and mitochondrial Ca^2+^ uptake [33]. ICCs, functioning as pacemakers, possess unique ionic conductances that trigger slow wave activity [34,35]. The synaptic-like contacts between nerve terminals and intramuscular ICCs facilitate the rapid diffusion of transmitters, further influencing the propagation of slow waves [31]. Thus, slow waves from ICCs ultimately control the contraction and relaxation cycles of smooth muscles in the gastrointestinal tract.

ICCs express c-kit; therefore, immunohistochemistry using an anti-KIT antibody is an effective and widely used method to identify them [27,36]. It was previously thought that ICCs also exist in various smooth muscle organs, other than the gastrointestinal tract. However, many reports of ICCs other than those of the gastrointestinal tract have not been double-stained with tryptase, and it is now possible that most of these reports were mast cells in tissues expressing c-kit [37]. Therefore, PICs are believed to be the main type of interstitial cells involved in the regulation of smooth muscle movement in smooth muscle organs other than the gastrointestinal tract [37,38].

## 3. Immunohistological Features of PICs

The PDGFR is a type III receptor tyrosine kinase expressed in various organs, including the GI tract [39]. It consists of two subtypes, PDGFRα and PDGFRβ, and has four ligands (PDGF-A, -B, -C, and -D) [40,41]. In the developmental stage of mammals, PDGF–PDGFR signals are crucial for organogenesis, including alveogenesis, hair morphogenesis, spermatogenesis, oligodendrogenesis, palate genesis, angiogenesis, and glomerulogenesis [39,42]. In mature organs, PDGFRα is known to be distributed in several interstitial-type cells such as cardiac fibroblasts, alveolar fibroblasts, dermal fibroblasts, hepatic stellate cells, and renal mesangial cells [39].

As mentioned above, PDGFRα is not specific to PICs and is also expressed on other types of fibroblasts. Therefore, PICs cannot be identified using PDGFRα alone. PICs also express CD34 and vimentin in addition to PDGFRα [43,44,45]. Additionally, Dennis et al. reported that mouse bladder PICs express multiple canonical fibroblast markers, including Col1a2, CD34, LY6A, and PDGFRα, along with the ubiquitous fibroblast genes *Col15a1* and *Pi16*. These results suggest that PICs are a type of fibroblast [38]. In addition, as will be described later, PICs express small-conductance Ca(2+)-activated K(+) channel 3 (SK3), which is classified as member of a family of calcium-activated potassium channels based on functional characteristics [46,47].

Furthermore, the expression of these markers varies depending on tissue and localization. For example, interstitial cells in the upper lamina propria of the human bladder are positive for vimentin, α-smooth muscle actin, caveolin-1 and 2, PDGFRα, and non-phosphorylated and phosphorylated connexin 43 [45]. In contrast, interstitial cells in the lower lamina propria are positive for vimentin, CD34, and unphosphorylated connexin 43 but negative for α-smooth muscle actin, caveolin-1 and 2, PDGFRα, and phosphorylated connexin 43 [45]. Therefore, heterogeneous immunohistochemical characteristics across animal species and tissues make PIC identification with molecular markers difficult.

## 4. Morphological Features of PICs

Electron microscopy observations of PICs reveal a distinct morphology characterized by a well-developed and rough endoplasmic reticulum, often featuring extended cisternae, Golgi apparatus, and mitochondria primarily located in the perinuclear region. Notably, these cells lack caveolae and basement membranes [48]. However, the qualitative nature of their morphological features, as observed via transmission electron microscopy (TEM), presents challenges in quantitative morphological characterization due to heterogeneity influenced by the local microenvironment [49].

To address this limitation, recent morphological studies have employed three-dimensional (3D) reconstruction techniques such as serial block-face scanning electron microscopy (SBF/SEM) and focused ion beam scanning electron microscopy (FIB/SEM) [50,51,52]. Mantani et al. utilized SBF/SEM to demonstrate that type III fibroblast-like cells, analogous to PICs in the lamina propria of the rat ileum, exhibit thin, mesh-like cell bodies and thinly branched cell processes immediately beneath the epithelium [49]. Similarly, Takeya et al., employing FIB/SEM, illustrated that PICs in guinea pig seminal vesicles possess sheet-like cell bodies with thicknesses ranging from 0.1 to 0.5 μm, along with sheet-like broad cell processes that overlap selectively [53]. Furthermore, Neuhans et al., utilizing both SBF/SEM and FIB/SEM, reported that PICs in the lamina propria of the human bladder exhibit sheet-like cell bodies and elongated cell processes, as revealed through 3D reconstruction [54]. A consistent morphological feature across these studies is the presence of very thin, mesh- or sheet-like cell bodies, contrasting with the spindle-like structures observed in conventional TEM-based 2D morphological analyses.

## 5. Functional Features of PICs

Neural regulation of smooth muscle movement in the gastrointestinal tract, an organ where functional studies of PICs have been conducted, involves both excitatory and inhibitory mechanisms. Excitatory mechanisms are primarily initiated by excitatory motor neurons releasing neurotransmitters like acetylcholine and substance P to enhance SMC excitability [55,56,57]. These neurotransmitters bind to muscarinic type 3 receptors and neurokinin 1 receptors expressed by ICCs [58,59]. Gq-coupled plasma membrane receptors activate phospholipase C (PLC), leading to the production of IP3 and diacylglycerol, which in turn trigger Ca^2+^ release and activate protein kinase C [60]. Diacylglycerol further amplifies this process by enhancing IP3 production through positive feedback on PLC activity [61]. The IP3-induced Ca^2+^ release is mediated by IP3 receptors (IP3Rs) in the endoplasmic reticulum (ER) [62]. The intense release of Ca^2+^ activates anoctamin 1 channels in the plasma membrane, coupled with IP3Rs [63], resulting in a depolarizing response that conducts to SMCs via gap junctions, ultimately leading to smooth muscle contraction.

In contrast, inhibitory mechanisms are mediated by inhibitory motor neurons through the release of neurotransmitters such as NO, ATP, β-nicotinamide adenine dinucleotide (β-NAD), and other purines [64,65,66]. ATP or β-NAD released from purinergic nerve terminals initially binds to P2Y1 receptors on PICs [47], which couple to G-protein Gq/11, activating PLC to increase intracellular IP3 [67]. IP3 stimulates Ca^2+^ release from intracellular calcium pools, activating SK3 channels and PICs, thereby causing cell hyperpolarization [47,68]. This results in the hyperpolarization of SMCs via gap junctions, leading to smooth muscle relaxation. Deep sequencing of gene transcripts in the small bowel and colonic PICs, purified by fluorescence-activated cell sorting (FACS), has revealed that these cells also express various receptors for additional neurotransmitters, hormones, and inflammatory mediators [69]. Growing evidence suggests that PICs act as a brake on gastrointestinal motility, integrating inhibitory inputs from intrinsic and extrinsic nerves, hormones, and inflammatory mediators [70]. Not only in the gastrointestinal smooth muscle but also in the detrusor smooth muscle, purines activate SK currents primarily via P2Y1 receptors in PICs [46,68].

## 6. Smooth Muscle Movement in Seminiferous Tubules

Spermatozoa lack active motility within the seminiferous tubules and are thus passively transported via testicular fluid from these tubules to the caput of the epididymis through the rete testis and efferent ductules. Although previous studies have noted bulk movement of luminal content [71,72], quantitative data on sperm transport within the seminiferous tubules are lacking. Earlier findings have suggested that contractile tubule movements are mediated by smooth-muscle-like testicular peritubular myoid cells (TPCs) [73,74]. Fleck et al. demonstrated that TPC contractions induce directional sperm movement within mouse seminiferous tubules both in vitro and in vivo [9].

TPCs encompass the seminiferous tubules in the mammalian testis. They exhibit contractile function and express cytoskeletal markers typical of smooth muscle, such as alpha-isoactin and smooth muscle myosin [75,76]. ATP [9], vasopressin [77], oxytocin [78], prostaglandins [79], and endothelin [80] have been identified as signaling factors that act on TPCs. ATP secretion from both Sertoli cells and germ cells occurs in a stimulus-dependent manner [81]. This secreted ATP activates TPCs through P2X and P2Y receptors, leading to seminiferous tubule contraction. However, the detailed mechanism underlying tubule contraction remains largely unknown.

## 7. PICs in Seminiferous Tubules

Morphological evidence supporting the presence of PICs surrounding the seminiferous tubules has been documented in humans [82], mice [83], rats [84], and Chinese soft-shelled turtles [85]. Immunohistochemistry and double immunofluorescence analyses revealed the co-expression of CD34, while testing negative for vimentin, α-SMA, c-kit, and CD31 [82,84,85]. PICs were observed outside the layer of TPCs surrounding the seminiferous tubules, characterized by a cytoplasm and a small cell body containing mitochondria around the nucleus. Adjacent PICs exhibited physical contact and formed a network connecting the TPCs, Leydig cells, and blood vessels [82,84,85]. Additionally, secretory vesicles were noted between PICs [82,84,85], suggesting the potential for direct cell conjugation, vesicle release, and paracrine and/or autocrine signaling.

As previously mentioned, while morphological studies on testicular PICs have been recently reported, functional studies remain absent. It remains unclear whether these cells express P2X and P2Y receptors; however, given the possibility of seminiferous tubule contraction via purinergic signaling [9,81], it is highly probable that PICs do indeed express P2X and P2Y receptors and play a role in seminiferous tubule contraction. Moreover, they may interact with Leydig and myoid cells, as well as blood vessels, potentially facilitating the transport of substances such as testosterone, crucial for spermatogenesis, to and from the seminiferous tubules.

## 8. Smooth Muscle Movement in Epididymal Ducts

Reabsorption of luminal fluid resulting from epithelial secretions and movements of ciliated cells may contribute to the transport of spermatozoa, but transportation mainly relies on the contractile activity of the SMC layer surrounding the epididymal epithelium [10,86,87]. However, actual sperm transport as a result of SMC contractility has only occasionally been reported.

The epididymal duct is characterized by spontaneous rhythmic contractions which decrease in frequency but increase in amplitude from head to tail, as shown in vivo [88,89,90] and in vitro [91,92,93]. There is also a report that the distal portion of the tail, which stores sperm until ejaculation, only sporadically contracts but does not spontaneously contract [94,95]. Additionally, adrenergic nerves are present in the caudal region, and it is believed that smooth muscle movement is mainly controlled by nerve input [10,96].

Spontaneous contractions in the epididymis are myogenic, suggesting the presence of pacemaking cells such as the ICC. Mewe et al. reported that cells isolated from bovine epididymal tracts exhibit three morphologically distinct contractile cell types: typical smooth muscle cells in the tail, myofibroblast-like cells along the ducts, and atypical myocytes with filaments. These cell types also demonstrated distinct biophysical properties. Atypical myocytes with filaments are speculated to provide electrical coupling between myofibroblasts, which is essential for the generation of regular myogenic activity [97].

Regarding the control of smooth muscle movement, it has been reported that not only neural input but also hormones, such as testosterone [98,99] and estrogen [100], as well as the influence of epithelial [101], sperm, and luminal factors [97], play a role. Cyclic guanosine monophosphate (cGMP) signaling plays a crucial role in the relaxation of SMCs in the epididymis [101]. This relaxation is mediated by the activation of cGMP-dependent protein kinase I and the subsequent inhibition of myosin light chain phosphorylation [102,103].

The involvement of purine signaling in the epididymis is also significant, with P2X1 and P2X2 receptors identified in the SMC layer, suggesting a role in regulating epididymal contractility and sperm ejection [104]. Additionally, the activation of P1 and P2 purinergic receptors by ATP and adenosine is proposed to play a role in luminal acidification, a crucial process for sperm maturation and storage [105].

## 9. PICs in Epididymis

**Figure 1:**
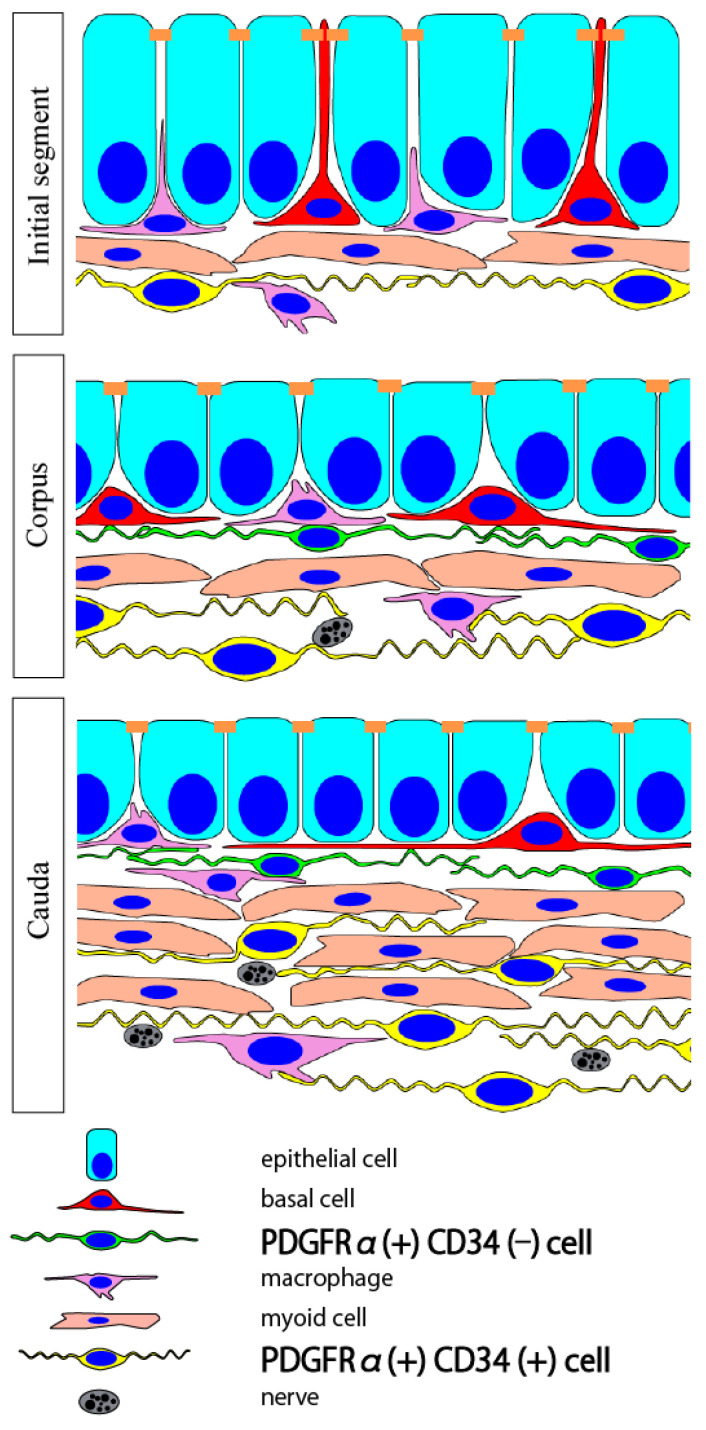
Schematic model of PIC subtypes present in the interstitial space of the murine epididymis. In the initial segment, 1–2 layers of CD34(+) PICs are present. In the other segments, the number of CD34(+) PIC layers increases toward the cauda. In the cauda, CD34(+) PICs are present between smooth muscle bundles. CD34(−) PICs are present beneath the epithelium in the other segments but not in the initial segment. PIC; PDGFRα-positive interstitial cell. Reprinted from [106] with permission of Elsevier, GmbH (Amsterdam, The Netherlands).

We have identified PICs in the epididymis of mice (Figure 1) [106]. They are classified into two subtypes, each possessing distinct immunohistochemical properties. The first subtype, located just beneath the epithelium, was CD34(−) and was observed from the corpus to the cauda but not in the initial segment (IS). The second subtype, CD34(+), was observed in the interstitial space, including the muscle layers of all segments. Furthermore, the density of CD34(+) PICs increased from the IS to the tail to match the thickness of the smooth muscle layer. Nerve fibers in the epididymis closely associated with CD34(+) PICs, which were observed both within and outside the smooth muscle layer. Therefore, CD34(+) PICs are presumed to interact with nerves, while CD34(−) PICs beneath the epididymal epithelium may be involved in transmitting information from the epithelium to the smooth muscle.

**Figure 2:**
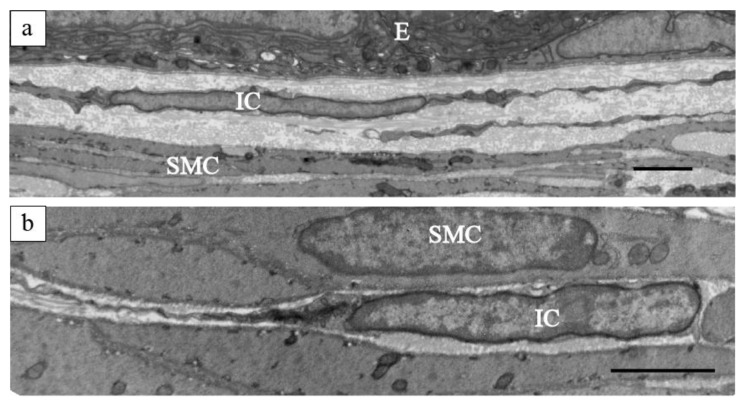
Ultrastructural images of PICs in the epididymal duct. (**a**) A PIC beneath the epithelium; (**b**) A PIC in the smooth muscular layer. IC, PDGFRα-positive interstitial cells; E, epithelium; SMC, smooth muscle cells. Reprinted from [106] with permission of Elsevier, GmbH. Scale bars: 10 μm (**a**), 500 nm (**b**).

TEM analysis revealed that the PICs in the murine epididymis lacked a basement membrane, exhibited a small amount of perinuclear cytoplasm, and displayed elongated cell processes (Figure 2) [106]. These ultrastructural characteristics closely resembled those of PICs previously described in other organs. However, no apparent differences were observed in the two-dimensional ultrastructure of the two PIC subtypes distinguishable by immunohistochemical features. Future research employing three-dimensional observations may uncover morphological disparities between these PIC subtypes.

**Figure 3:**
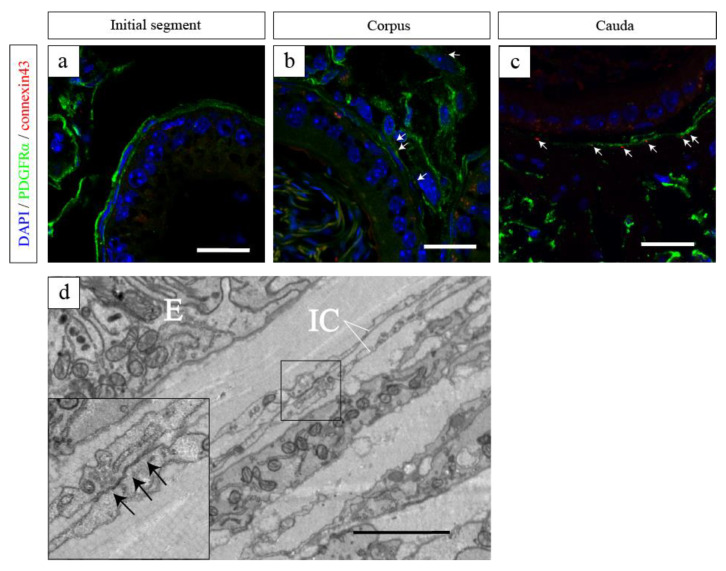
Representative images of the initial segment (**a**), corpus (**b**), and cauda (**c**) with double immunostaining for PDGFRα (green) and connexin 43 (red) [106]. (**a**–**c**) Nuclei were counterstained in blue with 4′,6-diamidino-2-phenylindole (DAPI). Co-localization PDGFRα and connexin 43 (white arrows of (**b**,**c**)). (**d**) Ultrastructural findings of PICs in the cauda. A close proximity area between cellular processes of PICs. High magnification of the black line square area of (**d**) (inset of (**d**)). An electron-dense region proximal to the PICs, indicative of a gap junction (black arrows, inset of (**d**)). IC, PDGFRα-positive interstitial cell; E, epithelium. Reprinted from [106] with permission of Elsevier, GmbH. Scale bars: 20 µm (**a**–**c**), 2 μm (**c**).

Connexin 43 was expressed in PICs spanning from the corpus to the cauda. Electron microscopy unveiled an electron-dense region proximal to the PICs, indicative of a gap junction (Figure 3) [106]. This observation suggests the potential existence of electrical interconnections among PICs within the epididymis through these gap junctions. Furthermore, their adjacency to nerves and smooth muscle implies a possible role in mediating smooth muscle contraction through physical interaction within the epididymis.

**Figure 4:**
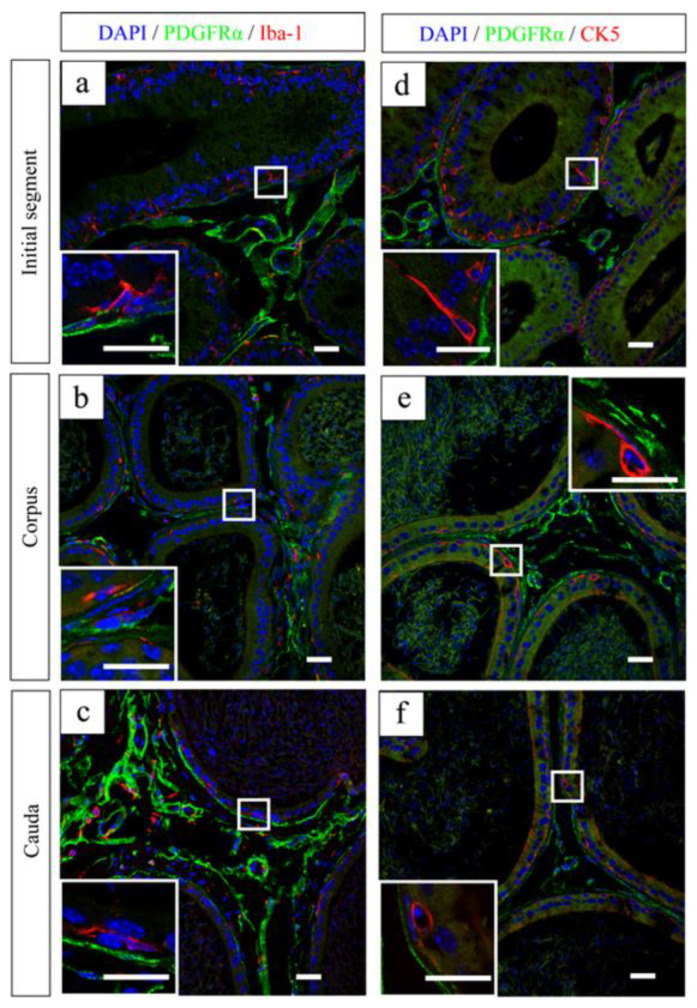
Representative images of the initial segment (**a**), corpus (**b**), and cauda (**c**) with double immunostaining for PDGFRα (green) and ionized calcium-binding adaptor molecule 1 (Iba-1; red). Nuclei were counterstained in blue with 4′,6-diamidino-2-phenylindole (DAPI). Representative images of the initial segment (**d**), corpus (**e**), and cauda (**f**) with double immunostaining for PDGFRα (green) and cytokeratin 5 (CK5; red) [106]. (**a**–**f**) Insets in (**a**–**f**): Higher magnification of the white square areas in (**a**–**f**). Reprinted from [106] with permission of Elsevier, GmbH. Scale bars: 20 µm.

CD34(−) PICs situated directly beneath the epithelium were closely associated with basal cells and macrophages (Figure 4) [106]. Macrophages localized near the epididymal duct are hypothesized to play dual roles: maintaining the integrity of the epithelial barrier and facilitating peripheral tolerance to auto-antigenic spermatozoa during maturation [107,108]. Simultaneously, they may also engage in immune surveillance, combating and monitoring pathogens that pose constant threats to the reproductive tract. Additionally, basal cells within the epididymis have been identified as luminal sensors regulating the activities of principal and clear cells, while also exhibiting scavenger functions [109,110]. Consequently, CD34(−) PICs may participate in various epididymal functions beyond sperm transport, including immunoregulation or epithelial activities.

## 10. Smooth Muscle Movement in the Vas Deferens

Sperm within the vas deferens are primarily transported through smooth muscle contraction. While numerous studies have investigated smooth muscle movement in the vas deferens compared to the seminiferous tubules and epididymis, relatively fewer studies have been conducted on the latter. The smooth muscle of the vas deferens is primarily regulated by autonomic nerves, with adrenergic nerves being the predominant group of nerve fibers supplying the vas deferens in mammals [111,112,113,114]. Research across various species has identified two distinct components of neurogenic contraction in the vas deferens. The first component involves rapid phasic contractions triggered by ATP acting on P_2X_ receptors, while the second component entails slower tonic contractions induced by noradrenaline and is independent of membrane depolarization [115,116,117,118]. These findings are corroborated by studies demonstrating the role of ATP and noradrenaline as co-transmitters in the vas deferens [116,118]. The initiation of these contractions is correlated with the rate and degree of depolarization, with phasic contraction being more sensitive to a reduction in extracellular calcium [119]. The selective blockade of ATP, noradrenaline, and electrically evoked contractions by nifedipine further underscores the involvement of ATP in contractile responses [120]. In the human vas deferens, Amobi et al. demonstrated that the stimulation of P2X1 purinoceptors elicited an excitatory effect leading to longitudinal muscle contraction [121], subsequently activating 4-aminopyridine-sensitive (KV) and iberiotoxin-sensitive (BKCa) K^+^ channels [122].

ATP acting on P2X1 receptors stimulates an influx of Ca^2+^ to produce excitatory junctional potentials (EJPs) that summate to depolarize the membrane sufficiently to activate VOCs [123]. The entry of Ca^2+^ then activates ryanodine receptors (RYRs) to release Ca^2+^ from the internal store. The initial release occurs proximal to the plasma membrane and subsequently propagates intracellularly through the regenerative release of Ca^2+^ by the RYRs and/or via inositol 1,4,5-trisphosphate (InsP3) receptors, manifesting as an intracellular Ca^2+^ wave [124]. The Ca^2+^ near the membrane activates large-conductance Ca^2+^-sensitive K^+^ (BK) channels and the resulting spontaneous transient outward currents hyperpolarize the membrane and help to terminate the activation process [125,126]. Noradrenaline (NA) acts by stimulating α1-adrenoreceptors to produce InsP3, which then releases Ca^2+^ that may induce an intracellular Ca^2+^ wave similar to that triggered by the ATP-dependent entry of external Ca^2+^. In addition, the α1-adrenoreceptors also activate the smooth muscle Rho/Rho kinase signaling pathway that serves to increase the Ca^2+^ sensitivity of the contractile machinery [127].

In vivo, there is also evidence suggesting that P2X1 receptors mediate postzygotic excitatory responses to ATP in the vas deferens and play a role in male reproductive function. Vas deferens tissue from P2X1 receptor-deficient (P2X1 receptor −/−) mice failed to respond to exogenously applied ATP or α,β-meATP, and these tissues lacked spontaneous and evoked EJPs [128]. This genetic deletion resulted in a 90% reduction in male fertility, attributed to a decreased spermatozoa count in ejaculated semen.

## 11. PICs in Vas Deferens

In the murine vas deferens, PICs are widely distributed across the lamina propria, smooth muscle, and serosal layers [129]. They have been categorized into two types, similar to those found in the epididymis: PDGFRα(+) CD34(−) PICs located just below the epithelium and PDGFRα(+) CD34(+) PICs observed within the interstitial space containing the muscle layer. PDGFRα(+) CD34(−) PICs exhibit a gradual increase in number from the testis side of the vas deferens, characterized by its thin smooth muscle layer, toward the urethra side, which features a thicker smooth muscle layer. The escalated distribution of PICs in both the epididymis and vas deferens, relative to the thickness of the smooth muscle layer, suggests their involvement in smooth muscle movement.

**Figure 5:**
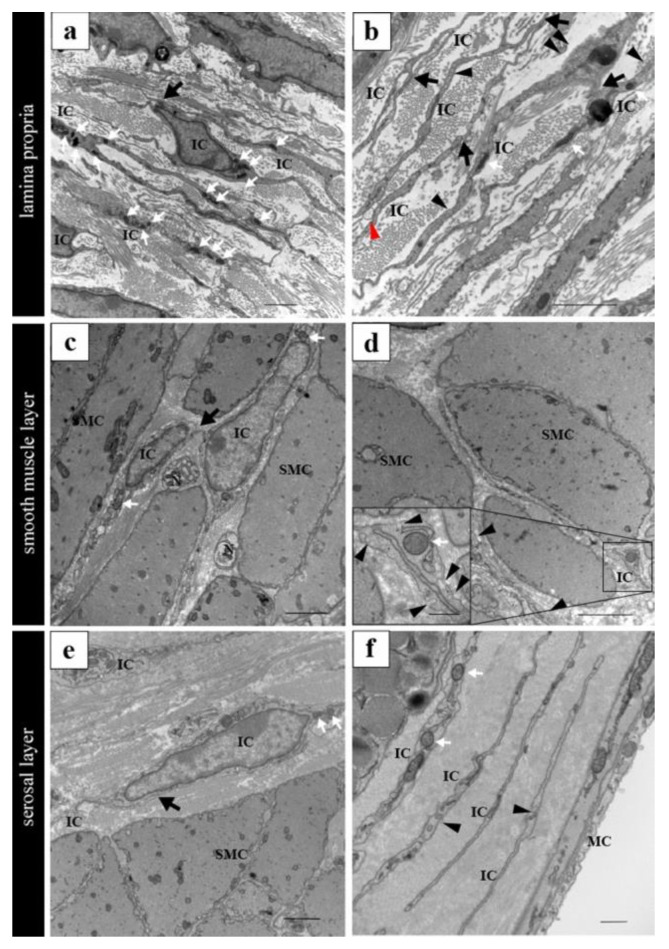
Ultrastructural findings in PICs of murine vas deferens. (**a**) PICs in the lamina propria of the vas deferens. (**b**) Cell processes of PICs in the lamina propria. Exosome-like structure between cell processes of PICs (red arrowhead). (**c**) PICs in the smooth muscle layer of the vas deferens. (**d**) Cell processes of PICs in the smooth muscle layer. High magnification of the solid black line square area (inset at the lower left corner of (**d**)). (**e**) PICs in the serosal layer of the vas deferens. (**f**) Cell processes of ICs in the serosal layer. PICs’ cell bodies are relatively small, with sparse cytoplasm containing several mitochondria surrounding the nucleus (white arrows). PICs were in close proximity to each other (black arrows). Caveolae in the cytoplasm of PICs in all the layers (black arrowheads). IC, PDGFRα-positive interstitial cells; SMC, smooth muscle cells; MC, mesothelial cells. Images shown are taken from a paper [129]. Scale bars: 2 μm (**a**–**e**); 500 nm ((**f**), inset at the lower left corner of (**d**)).

**Figure 6:**
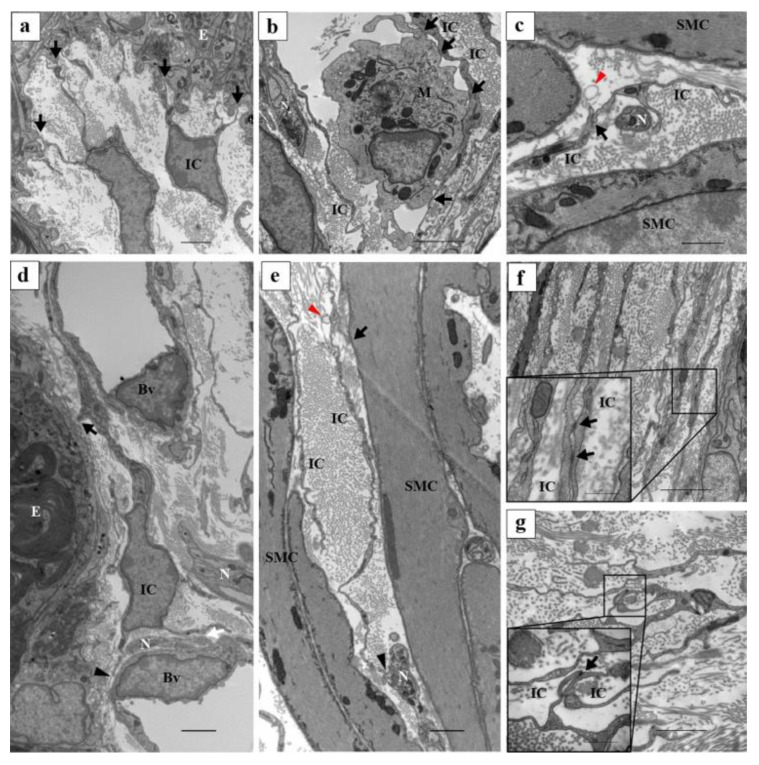
Ultrastructural findings showing the relationship between PICs and surrounding cells in murine vas deferens. (**a**) The cell processes of the ICs were in close proximity to the epithelium (black arrows). (**b**) A cell process of the IC was in close proximity to a macrophage (black arrows). A cell process of the IC wraps nerves and is in close proximity to the cell process of the other IC (black arrows). (**c**) Exosome-like structure near the cell process of the IC (red arrowhead). (**d**) The ICs in the lamina propria were in close proximity to surrounding tissues. The IC in the lamina propria was in close proximity to the epithelial cell (black arrow), a vascular endothelial cell (black arrowhead), and a nerve (white arrow). (**e**) The IC in the smooth muscle layer was in close proximity to the surrounding tissues. The IC in the smooth muscle layer was in close proximity to a smooth muscle cell (black arrow) and a nerve (black arrowhead). Exosome-like structure near the cell processes of the IC (red arrowhead). (**f**,**g**) TEM images of contact areas between ICs. Higher magnification of the solid black line square area (inset at the lower left corner of (**f**,**g**)). Electron-dense lines exist in the contact area between ICs (black arrows). ICs, interstitial cells; E, epithelium; N, nerves; Bv, blood vessels; M, macrophages. Images shown are taken from a paper [129]. Scale bars: 2 μm (**a**–**g**); 500 µm (inset at the lower left corner of (**f**,**g**)).

The cell bodies of PICs appear relatively small, with sparse cytoplasm containing some mitochondria observed around the nuclei (Figure 5a–f). Notably, a basement membrane is absent, while several elongated cell processes are evident, facilitating proximity among PICs (Figure 5a–c). Additionally, a few caveolae are discernible in the cytoplasm (inset of Figure 5d). Connexin 43 expression is notable in PICs, with gap-junction-like structures observed via electron microscopy (Figure 6f,g). Morphologically, PICs within the vas deferens exhibit electrical connections with each other and are closely situated near nerves and smooth muscles (Figure 6c,e). Furthermore, PICs within the lamina propria are in close proximity not only to nerves and smooth muscle but also to the epithelium (Figure 6a,d), blood vessels (Figure 6d), and macrophages (Figure 6b).

**Figure 7:**
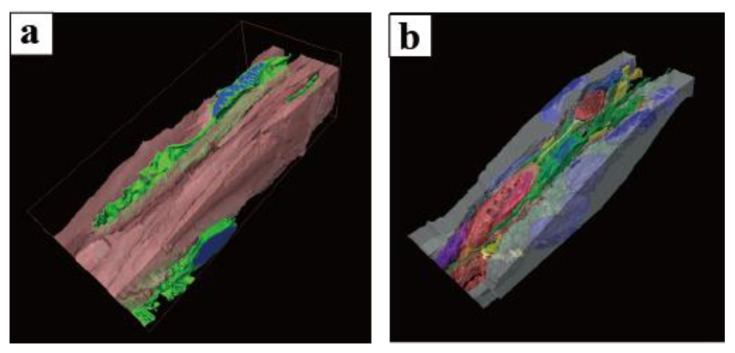
High-magnification images within the murine vas deferens for which a series of sequential images were acquired using FIB/SEM: (**a**) smooth muscle layer; (**b**) lamina propria. The object surfaces are color-coded: PDGFRα-positive interstitial cells, light green; nuclei, blue; smooth muscle cell, coral; epithelium, grey; vessels, red; nerves, yellow. The same images following deconvolution and 3D reconstruction using the Avizo software (version 9.1.1) are available at https://www.fei.com/software/avizo3d/ (last accessed date: 2 March 2024). Reprinted from [130,131] with permission of Elsevier, GmbH.

A 3D reconstruction of PICs in the vas deferens, performed using FIB/SEM, revealed that PICs possess sheet-like structures rather than spindle-like structures, consistent with previous three-dimensional analyses of PICs in other organs (Figure 7a,b) [130,131]. In other words, the findings observed by TEM suggest a cross-section of a sheet-like cell body of PICs. Furthermore, the 3D reconstruction of PICs using FIB/SEM highlighted morphological differences between PICs in the lamina propria and those in the smooth muscle layer.

**Figure 8:**
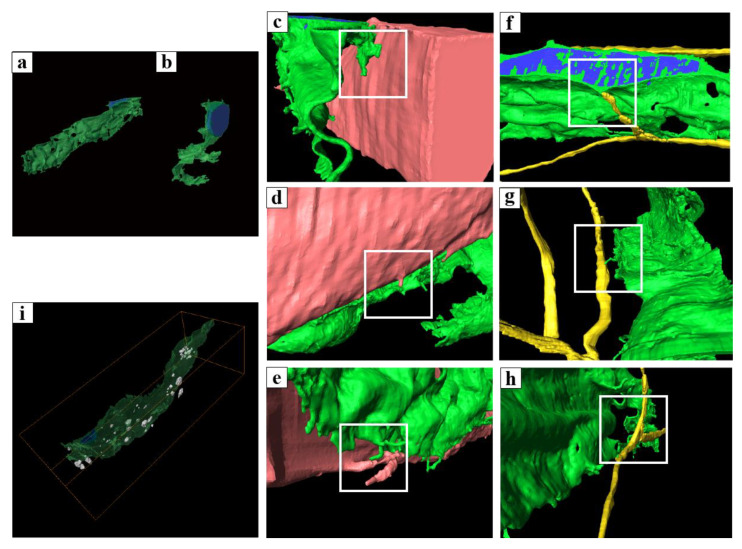
3D reconstructed image of PICs in the smooth muscle layer and 3D reconstruction of the interfaces between surrounding tissue and PICs. (**a**) Stereoscopic 3D reconstructed image of the flattened, sheet-like PICs. (**b**) Stereoscopic 3D reconstructed image of the curled, sheet-like PICs. (**c**–**e**) 3D reconstruction of the interfaces between smooth muscle cells and PICs (white squares). (**f**–**h**) 3D reconstruction of the interfaces between nerves and PICs (white squares). (**h**) 3D reconstruction of a PIC and the exosome-like structures around it. Object surfaces are color-coded: PICs, light green (**a**–**h**), transparent green (**i**); nuclei, blue; smooth muscle cell, coral; nerves, yellow; exosome-like structures, grey. The same images following deconvolution and 3D reconstruction using the Avizo software (version 9.1.1) are available at https://www.fei.com/software/avizo3d/ (last accessed date: 2 March 2024). Reprinted from [130] with permission of Elsevier, GmbH.

PICs in the smooth muscle layer had two types of 3D sheet-like structures: flat and curled (Figure 8a). Various spatial relationships have been observed between PICs, neurons (Figure 8b–d), and smooth muscle cells (Figure 8e–g), which form a complex 3D. In addition, many extracellular vesicle-like structures were observed around PICs. Since the sheet-like structure increases cell surface area and is a very effective structure for the delivery of extracellular vesicles and for physical interactions and exchange of humoral factors by such vesicles, PICs may be involved in neuromuscular signal transduction (Figure 8h).

**Figure 9:**
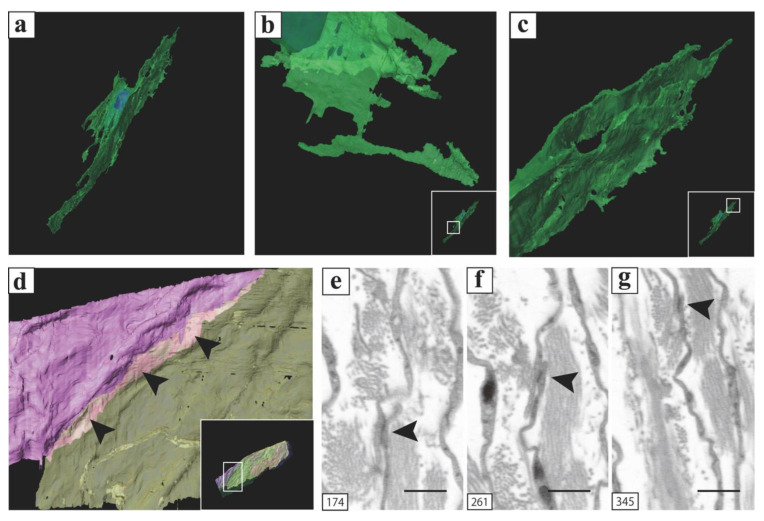
3D reconstructed image of PICs in the lamina propria and 3D reconstruction of the interfaces between PICs [131]. (**a**) 3D reconstructed image of a flattened, sheet-like PIC with multiple cellular processes. (**b**) Higher magnification of the flattened, sheet PIC processes. (**c**) Higher magnification of the elongated, rod-shaped PIC processes. (**d**) Higher magnification of the white square area in the inset showing the 3D reconstruction of interfaces between PICs (black arrows). (**e**–**g**) 2D digital slices including the areas indicated by black arrowheads in (**d**) extracted from the sequential images. High electron density was observed in the areas indicated by black arrows in (**d**) (black arrows in (**e**–**g**)). Images shown are taken from a paper [131].

The PICs in the lamina propria exhibited a flat, sheet-like structure with cytoplasm and multiple cellular processes (Figure 9a). Additionally, two types of 3D structures comprising cell processes were observed: a rod-shaped structure (Figure 9b) and an accordion-fold-like one (Figure 9c). PICs were positioned parallel to the epithelium (Figure 9d) and were interconnected through gap junctions or adherens junctions (Figure 9e–g). Notably, PICs in the lamina propria were folded akin to a screen between the epithelial basement membrane and the smooth muscle, suggesting their potential involvement in regulating smooth muscle movement by the epithelium.

## 12. Conclusions

To date, numerous morphological studies on interstitial cells have been reported. These studies represent the initial step in elucidating their functional roles, given that the characteristic morphology of these cells may influence smooth muscle movement. The morphological investigations of PICs in male reproductive organs offer intriguing possibilities, indicating that PICs might participate in sperm transport mechanisms within the male reproductive tract. Further functional research on PICs in the male reproductive tract holds the potential for a breakthrough in ejaculatory disorders, for which effective therapeutic strategies have not yet been established.
